# Central effects of soluble factors derived from human macrophages, polarized to M2 phenotype, in depression

**DOI:** 10.1192/j.eurpsy.2025.1692

**Published:** 2025-08-26

**Authors:** E. Markova, E. Serenko, M. Knyazheva, E. Shevela, M. Tikhonova, T. Amstislavskaya

**Affiliations:** 1 Neuroimmunology Lab; 2 Cellular Immunotherapy Lab, State Research Institute of Fundamental and Clinical Immunology; 3 Neurobiological mechanisms of neurodegenerative processes Lab; 4 Experimental models of neuropsychiatric disorders Lab, State Research Institute of Neurosciences and Medicine, Novosibirsk, Russian Federation

## Abstract

**Introduction:**

In depressive disorders caused by chronic psychological stress, cognitive decline is produced by neuroinflammatory and neurodegenerative changes in the brain. M2-type macrophages possess high pronounced regenerative potential due to high production of neurotrophic, neuroprotective and angiogenic factors. We have previously shown that M2 macrophage-derived soluble factors (M2-SFs) edit stress-induced depressive-like behavior.

**Objectives:**

The aim of the study was to investigate the central effects of soluble factors derived from human macrophages, polarized to M2 phenotype (M2-SFs) involved in the mechanisms underlying the editing of depressive-like behavior.

**Methods:**

Human macrophages were polarized polarised to M2 phenotype under serum deprivation conditions. Stress-induced depression-like male mice were undergoing intranasal administration of M2-SFs during 7 days. After which an immunohistochemical analysis was performed assessing the expression of the microglial marker Iba1. The levels of brain-derived neurotrophic factor (BDNF) and cytokines in separate structures of the brain were assessed by ELISA. For histological examination Nissl staining was applied.

**Results:**

Depressive-like behavior editing after the M2-SFs administration was registered against the background of some structural and functional changes in the brain. It was found an increase in the density of pyramidal neurons in the frontal cortex and augmented level of BDNF in this brain structure and also in the hippocampus. After the introduction of the M2-SFs in depressive-like mice the decreased expression of the microglial marker Iba1, accompanied with decreased levels of pro-inflammatory cytokines IL-1β, IL-6, TNF-α, INF-γ in pathogenetically significant structures of the brain was also observed.

**Conclusions:**

The data obtained indicate that the depressive-like behavior-editing effect of M2 macrophage-derived soluble factors is mediated by stimulating neurogenesis, neuroplasticity and reducing neuroinflammation.

**Disclosure of Interest:**

None Declared

